# D-Bifunctional Protein Deficiency Diagnosis—A Challenge in Low Resource Settings: Case Report and Review of the Literature

**DOI:** 10.3390/ijms25094924

**Published:** 2024-04-30

**Authors:** Maria Livia Ognean, Ioana Bianca Mutică, Gabriela Adriana Vișa, Ciprian Radu Șofariu, Claudiu Matei, Bogdan Neamțu, Manuela Cucerea, Radu Galiș, Gabriela Ariadna Cocișiu, Ioana Octavia Mătăcuță-Bogdan

**Affiliations:** 1 Faculty of Medicine, Lucian Blaga University, 550025 Sibiu, Romania; maria.ognean@ulbsibiu.ro (M.L.O.); matei.claudiu@ulbsibiu.ro (C.M.); bogdan.neamtu@ulbsibiu.ro (B.N.); ioana.matacutabogdan@ulbsibiu.ro (I.O.M.-B.); 2 Neonatology Department, Clinical County Emergency Hospital, 550245 Sibiu, Romania; 3 Research and Telemedicine Center in Pediatric Neurology, Pediatric Clinical Hospital Sibiu, 550169 Sibiu, Romania; visa.gabriela@gmail.com (G.A.V.); ciprianradusofariu@gmail.com (C.R.Ș.); 4 Pediatric Clinical Hospital Sibiu, 550169 Sibiu, Romania; 5 Department of Computer Science and Electrical Engineering, Faculty of Engineering, Lucian Blaga University Sibiu, 550025 Sibiu, Romania; 6 Department of Neonatology, George Emil Palade University of Medicine, Pharmacy, Science, and Technology, 540142 Targu Mures, Romania; manuela.cucerea@umfst.ro; 7 Department of Neonatology, Clinical County Emergency Hospital Bihor, 410167 Oradea, Romania; radu.galis@scjubh.ro; 8 Department of Neonatology, Poznan University Medical Sciences, 60-512 Poznan, Poland; 9 Medical Laboratory, Military Emergency Hospital, 550024 Sibiu, Romania; laborator@spitalmilitarsb.ro

**Keywords:** peroxisomal disorders, D-bifunctional protein deficiency, whole-exome sequencing, neonatal hypotonia, neonatal seizures

## Abstract

D-bifunctional protein deficiency (D-BPD) is a rare, autosomal recessive peroxisomal disorder that affects the breakdown of long-chain fatty acids. Patients with D-BPD typically present during the neonatal period with hypotonia, seizures, and facial dysmorphism, followed by severe developmental delay and early mortality. While some patients have survived past two years of age, the detectable enzyme activity in these rare cases was likely a contributing factor. We report a D-BPD case and comment on challenges faced in diagnosis based on a narrative literature review. An overview of Romania’s first patient diagnosed with D-BPD is provided, including clinical presentation, imaging, biochemical, molecular data, and clinical course. Establishing a diagnosis can be challenging, as the clinical picture is often incomplete or similar to many other conditions. Our patient was diagnosed with type I D-BPD based on whole-exome sequencing (WES) results revealing a pathogenic frameshift variant of the *HSD17B4* gene, *c788del*, p(Pro263GInfs*2), previously identified in another D-BPD patient. WES also identified a variant of the *SUOX* gene with unclear significance. We advocate for using molecular diagnosis in critically ill newborns and infants to improve care, reduce healthcare costs, and allow for familial counseling.

## 1. Introduction

Peroxisomal diseases encompass a diverse group of uncommon genetic metabolic conditions, affecting approximately 1 in 25,000–50,000 newborns [1,2,3,4,5]. These conditions may result from defects in peroxisomal biogenesis, such as the Zellweger spectrum syndrome, or deficiencies in key enzymes or transporters involved in the peroxisomal metabolism [3,4,6,7,8]. The most prevalent peroxisomal enzymatic deficit is D-bifunctional protein deficiency (D-BPD) (OMIM #261515), an autosomal recessive neurodegenerative disorder classified as single a enzyme peroxisomal disorder [1,9]. D-bifunctional protein (D-BP), a steroid-metabolizing enzyme, has three active domains—N-terminal dehydrogenase, a central hydratase, and a C-terminal steroid carrier protein 2-like domain—involved in the peroxisomal beta-oxidation of specific fatty acids and the synthesis of bile acids [1,5]. The D-BP is involved in the second (hydration) and third step (dehydrogenation) of the peroxisomal fatty acid β-oxidation pathway (very long fatty acids, branched fatty acids, and bile acid intermediates’ cleavage) [9]. D-bifunctional protein deficiencies are divided according to the degree of activity of the hydratase and dehydrogenase protein units [6]. The disease is considered an inborn error of peroxisomal oxidation, affecting various peroxisomal substrates (e.g., very-long-chain acyl-CoAs and bile acid precursors) in individuals with homozygous or compound heterozygous mutations in the *HSD17B4* gene (17 β-hydroxysteroid dehydrogenase type IV) [3,4,9,10,11]. More than 83 mutations of the *HSD17B4* gene have been described in humans [12], followed by structural changes and total or partial loss of the enzymatic function of D-BP [1]. The first case was described in 1989 by Watkins et al. [13] as L-protein deficiency, and following the *HSD17B4* gene’s discovery and description, the cases were re-evaluated and classified as D-BPD in 1999.

D-bifunctional protein deficiency is a rare genetic disorder that affects newborns, with a prevalence of 1 in 30,000 to 1 in 100,000 [3,9,11]. Symptoms typically manifest during the neonatal period, including hypotonia, seizures, and facial dysmorphism [3,9,11,14,15]. The condition leads to severe psychomotor retardation and death within the first two years of life in most cases. Depending on deficient enzymatic activity, D-BPD is classified into four types: type I—defined by dehydrogenase and hydratase deficiency; type II—associated with only hydratase deficiency; type III—defined by dehydrogenase deficiency; type IV—described by McMillan [5], with a less severe phenotype compared to types I, II, and III, similar to Perrault syndrome (PRLTS) (OMIM #233400), another rare disease associated with mutations of the *HSD17B4* gene [3,5,6,7,12,16]. Recently, a fifth type, type V, was proposed, with clinical features that overlap with D-BPD and Perrault syndrome, defined by neurological manifestations, sensorineural hearing loss in both genders, and primary ovarian insufficiency in females [17].

The diagnosis of D-BPD is a multifaceted process, posing significant challenges in low-resource settings. It typically involves a series of steps, including clinical evaluation, laboratory analysis, and genetic testing. The clinical features, course of the disease, and imaging findings are similar to other peroxisomal disorders—D-BPD was initially named pseudo-Zellweger syndrome or Zellweger-like syndrome [3,4,7,18,19,20]. Peroxisomal markers of the disease, such as very-long-chain fatty acids (VLCFA), pristanic acid, phytanic acid, and biliary acids, may not be consistently increased [5,6,18,21]. Enzyme studies on skin fibroblast cultures are not universally available and can take time, but they allow for direct measurement of the enzyme [9].

Whole-genome and -exome sequencing are typically the last step in diagnosis. However, these tests can provide valuable information for patient care, including earlier diagnoses, outcome prediction, and family genetic counseling [5]. Genetic diagnosis methods, such as whole-genome sequencing (WGS) and whole-exome sequencing (WES), are accurate, rapid, available, and have recently become less expensive, making them an attractive option for diagnosing rare hereditary conditions [14,22,23]. Experts recommend WES and WGS as standard methods in situations where diagnosing rare diseases is difficult [14,24,25,26], particularly in areas with fewer specialized centers and specialists. The traditional approach, ~slow shut gun~, is slow and expensive as it incurs additional costs for hospitalization, follow-up in ambulatory settings, medication, treatment complications, and iatrogenic costs, additionally to the cost of the diagnostic tests, according to Soden et al., 2014 [27]. There is a growing call to integrate genomics into the continuous flow of clinical care [28,29,30], particularly in critically ill children [28].

We are reporting on the diagnosis of Romania’s first type I D-BPD case, caused by a homozygous frameshift mutation in the *HSD17B4* gene. Interestingly, this genetic defect was associated with another homozygous mutation in the sulfite oxidase (*SUOX*) gene, with uncertain significance. The *SUOX* gene is known to be involved in isolated sulfite oxidase deficiency (ISOD).

## 2. Results

The female newborn was delivered naturally in cranial presentation, with meconium-stained amniotic fluid at 40 weeks gestational age, with a birth weight of 2590 g (<3rd percentile), height 53 cm (90th percentile), head circumference of 33 cm (25th percentile), and Apgar score of 6, 8, and 9 at 1, 5, and 10 min, respectively, with a score of 1 for tone at every evaluation. After applying routine neonatal resuscitation procedures, the newborn seemed to adapt well to extrauterine life. However, at 3 h of life, the infant experienced desaturation episodes and required around 3 h of free-flow oxygen. Routine screening for hypoglycemia revealed a blood glucose level of 36 mg/dL, promptly corrected with oral glucose and feeding. Persistent hypotonia and weak archaic reflexes, including the feeding reflexes, were noted immediately after birth and persisted during the first days of life. Laboratory investigations showed increased values of the inflammatory markers; early onset neonatal sepsis with *Escherichia coli* was diagnosed and treated with intravenous antibiotics for 12 days. Feeding difficulties led to gavage feeding starting on the second day of life (DOL). The infant was admitted to the neonatal intensive care unit on the 3rd DOL as she presented apnea, a high-pitched cry, clonic movements, and lateral nystagmus, confirmed by amplitude electroencephalography (aEEG) as seizures. The infant was administered phenobarbital and topiramate to manage the seizures, along with oxygen on the nasal cannula and vitamin B6 for 9 days. However, the seizures reoccurred on the 12th day, accompanied by apnea and low oxygen saturation. The infant was on non-invasive respiratory support (continuous positive airway pressure (CPAP)) for 5 days, and we added levetiracetam to control the seizures. Despite continuous adjustments of anticonvulsant dosage, the infant presented various types of seizures several times a day, typically for a brief duration.

The routine biochemical tests, including proteins, lipids, glucose, liver and kidney function, blood gas analysis, lactates, and blood count with formula, were all within a normal range up to the 14th day of life; inflammatory markers were normalized by DOL 12 and afterward. Echocardiography at DOL 17 showed persistent ductus arteriosus and foramen ovale; abdominal and lung ultrasounds were normal. A head ultrasound on DOL 17 revealed the cystic transformation of the left caudate nucleus, mild ventriculomegaly, and lenticulostriate vasculopathy. These findings were interpreted in the context of chronic fetal distress, intrauterine growth restriction, and mild hypoxia at birth, and they persisted up to the 47th day of life.

The recurrence of seizures, persistent hypotonia, and hyporeflexia accompanied by feeding difficulties prompted the need for a re-evaluation of the etiology of neonatal seizures. Neurological evaluations also noted the gradual disappearance of osteotendinous reflexes and minimal spontaneous movements. The aEEG revealed low-voltage delta waves, with superimposed, biphasic, or triphasic sharp waves in the left anterior derivations.

Family history had no relevance regarding hereditary conditions: parents were young (mother, 27 years, father, 25 years), without consanguinity, and the pregnancy was incorrectly monitored (low social, economic, and educational status) but uneventful. There were no abortions or fetal losses, and the seven-year-old brother of the family was healthy. Metabolic screening for 18 amino acid disorders, 18 organic acids disorders, 13 fatty acids metabolism disorders, 7 other metabolic or endocrine disorders, tandem mass spectrometry for Pompe disease, and re-evaluation of the results of metabolic routine biochemical tests were all within normal limits. MLPA for spinal muscular atrophy was negative. Ophthalmological exams and auditive tests showed no abnormalities, just a delay in retinal vascularization compared to the infant’s gestational age. Except for a large anterior fontanelle, no abnormal features were noted that would allow for association with a genetic syndrome. Genetic sequencing for epilepsy (Illumina MySeq) using Illumina TruSight One for targeted amplification evaluated 186 genes associated with various epileptic phenotypes and identified no variants with pathogenic, possible pathogenic, or uncertain clinical significance.

**Figure 1:**
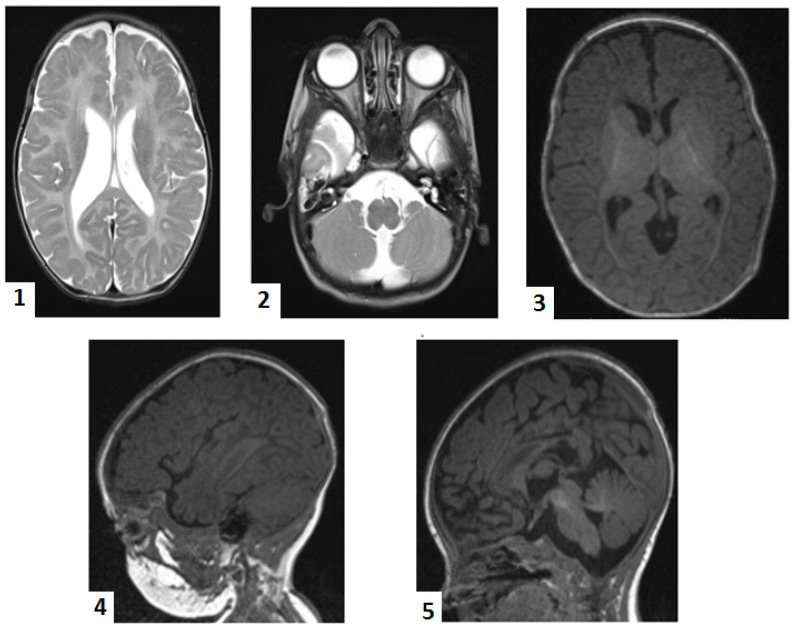
Magnetic resonance imaging: 1. enlarged ventricles; 2. bilateral temporal subarachnoid cyst, mega cisterna magna; 3. delayed partial myelination of the posterior limb of the internal capsule; 4. bilateral perisylvian polymicrogyria; 5. cerebellar atrophy (central lobule, culmen), dysgenesis of the corpus callosum.

At 2 months, brain magnetic resonance imaging (MRI) showed bilateral perisylvian polymicrogyria, delayed partial myelination of the posterior limb of the internal capsule, cerebellar atrophy (central lobule and culmen) with secondary mega cisterna magna, callosal dysgenesis, and bilateral temporal subarachnoid cysts (Figure 1). These findings led us to suspect a rare neurodegenerative disorder. We then performed a genetic test on the patient’s buccal swab using whole-exome sequencing (Blueprint Genetics Whole Exome Plus Test version 2, Espoo, Finland, 9 February 2018). The results showed that the patient had two homozygous mutations: HSD17B4 c.788del.p(Pro263GInfs*2) (NM_000141.4) and *SUOX c.913G>A, p.(Ala305Thr)(NM_000456.3)*. Upon searching in genetic databases (ClinVar, gnomAD, HGMD), it was found that the *HSD17B4* variant was previously reported once as pathogenic in ClinVar [10]. However, the *SUOX* variant, although previously reported once in the heterozygous variant in gnomAD, was predicted as deleterious by some in silico tools, including PolyPhen and MUTTASTER. Technical details on WES workflow are presented in the Supplementary Materials. We offered genetic counseling to the parents and stopped further biochemical confirmation of the D-BPD. This decision was taken as the extended metabolic screening and all of the genetic tests performed up to this moment were supported by a non-governmental charity organization. Given the lethal outcome, the parents refused other investigations.

It is important to note that there is no specific treatment for D-BPD. A deficiency of long-chain polyunsaturated fatty acids, particularly docosahexaenoic acid (DHA), has been associated with brain and retinal impairment [12]. However, despite increasing DHA levels, DHA supplementation had no benefit in treating the DHA deficit [31].

**Figure 2:**
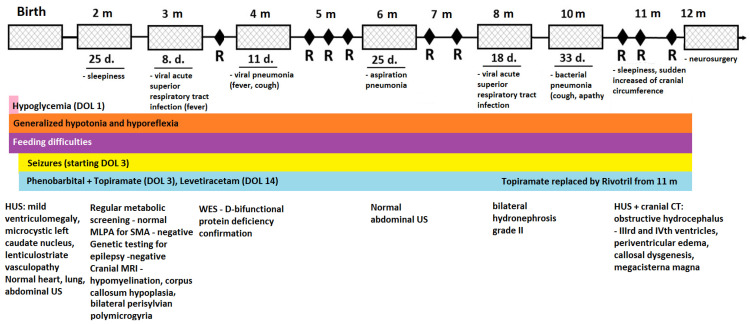
Clinical course, investigations, and follow-up of the proband. Legend: m—months; d.—days; R—outpatient re-evaluation; DOL—day of life; HUS—head ultrasonography; US—ultrasonography; MLPA—multiplex ligation-dependent probe amplification; SMA—spinal muscular atrophy; MRI—magnetic resonance imaging; WES—whole exome sequencing; CT—computer tomography.

The infant had an unfavorable clinical course, with recurrent respiratory tract infections leading to multiple hospital admissions and outpatient evaluations (Figure 2). The anticonvulsants’ dosages had to be adjusted several times. Hypotonia, hyporeflexia, and absent osteotendinous reflexes persisted over time. Feeding difficulties led to protein-caloric malnutrition and anemia despite high-calorie feedings and vitamin and iron supplementations. At 8 months, the infant’s developmental quotient was equivalent to a one-month-old’s. She had no control, limited fine and gross motor ability (hardly grasping and manipulating objects), nonverbal communication, and severely delayed impressive and expressive language. The infant demonstrated a weak cry, affective immaturity, and fluctuant behavior.

The infant presented various types of seizures almost daily, which required continuous adjustments of anticonvulsants doses and regimens. Following the neonatal period, the seizures predominantly became focal, initially every 2–3 days, increasing in frequency towards one year of age, when, despite treatment, occurred daily, associated with apnea. Conventional electroencephalography, performed in time, showed a disorganized pattern with a 2–3 Hz delta rhythm, variable amplitude (12–40 µV), and overlapping sharp-wave and polysharp-wave bilateral discharges, particularly in frontal and temporal derivations from various foci. Abdominal ultrasound performed at 8 months revealed bilateral hydronephrosis grade II. A sudden increase in the cranial circumference associated with sleepiness prompted the parents to seek an outpatient evaluation. The cranial computer tomography (CT) performed at this moment confirmed the ultrasound aspect of mixed-obstructive and secondary to white matter loss—hydrocephalus with periventricular edema. Callosal dysgenesis and megacisterna magna were also observed. The infant underwent ventriculoperitoneal shunting, which was uneventful. There was a short-term improvement in the neurological status, but the next day, almost continuous seizure activity imposed invasive ventilation. Recurrent bradycardia and asystole followed, and despite inotropic, vasoactive support and extensive resuscitation maneuvers, the infant passed away a few hours later.

## 3. Discussion

Peroxisomal disorders are a rare, heterogeneous group of neurodegenerative hereditary metabolic conditions broadly classified into two types: peroxisomal biogenesis defects and defects in a key enzyme in peroxisomal metabolism [3,4,7,8]. Peroxisomes are organelles present in all types of cells, found in various numbers and shapes; their enzymatic content depends on the organism, tissue, and even the environment [8,12]. The approximately 50 peroxisome proteins are involved in various catabolic and anabolic reactions [8]. In humans, two bifunctional proteins are described: protein L (or multifunctional protein 1) and D (or multifunctional protein 2), both of which are encoded by the *HSD17B4* gene [8,9].

D-bifunctional protein, discovered in 1996 [32], is a stereospecific steroid metabolizing peroxisomal enzyme that is largely distributed throughout the entire body, particularly in the liver [9,16]. D-bifunctional protein deficiency (OMIM #261515) is classified as a single-enzyme peroxisomal disorder with an interesting history. The genetic defect was first demonstrated in 1999 by van Grunsven et al. [33]. The first case was described in 1989 by Watkins et al. [13] as L-protein deficiency, which was later re-evaluated in 1999 and classified as D-BPD. Another two cases described by Suzuki et al. in 1994 as L-bifunctional protein deficiency were later, in 1997, identified as D-BPD [34]. Due to its clinical aspects, evolution, and severity, which are similar to Zellweger syndrome, D-BPD was initially named Zellwegger-like syndrome or pseudo-Zellweger syndrome [7,20].

### 3.1. Clinical Aspects and Investigations

In 2006, Ferdinandusse et al. [9,11] conducted a comprehensive review of D-BPD. The review covered various aspects of the disease, including its clinical features, diagnosis, disease progression, and outcomes. The authors evaluated the clinical and molecular data of 126 patients and identified 61 unique mutations causing D-BPD. Among these, 48 mutations were reported for the first time. Since then, rare cases of D-BPD have been reported, and genetic databases show that, as of 2023, there are 110 pathogenic variants in the *HSD17B4* gene in the Human Gene Mutation Database (HGMD), for example. The estimated prevalence of D-BPD is between 1 in 30,000 and 1 in 100,000 [9,11].

Ferdinandusse et al. [9,11] have documented a clinical pattern of the clinical aspect of D-BPD: virtually all infants in their cohort presented during the neonatal period with hypotonia (98%) and neonatal seizures (93%). In most cases, the infants did not show any developmental progress, or any motor abilities they initially showed were lost over time. The condition continued to progress into severe psychomotor delay and persistent seizures resistant to anticonvulsants, ultimately leading to death by the age of 2 years. Less severe cases are rare and may survive with gross motor delay, absent or severe language delay, and neurosensorial deficits [5,18]. Our patient presented with marked hypotonia and weak reflexes, including weak feeding reflexes since birth, which were persistent throughout her entire life. Seizures began in our patient in the third DOL and were initially managed using two anticonvulsants. However, they relapsed after 9 days and became progressively more difficult to manage as their frequency, duration, and resistance to anticonvulsants increased. Additionally, although osteotendinous reflexes were present in the first few days of life, they gradually disappeared. From the neonatal period, the infant had limited movements, and a comprehensive evaluation at 8 months showed almost no fine or gross motor abilities and severe language delay.

Despite the neonatal clinical picture, multiple factors delayed the final diagnosis in our case. Initially, the symptoms were thought to be caused by moderate birth hypoxia due to chronic fetal distress and early onset neonatal sepsis with *E. coli*. The infant received treatment accordingly. Only the reoccurrence of seizures, persistent hypotonia, and feeding difficulties pointed to another etiology and prompted a re-evaluation of the infant. The framing as a genetic condition was difficult as no physical abnormalities were observed apart from a large anterior fontanelle. Therefore, at that point, our investigations ruled out common metabolic disorders, infections, and malformations.

In the Ferdinandusse et al. cohort [9,11], 68% of the cases showed cranial and facial dysmorphisms. Other authors have also reported facial dysmorphisms, usually similar to Zellweger syndrome [2,7,11,16]. Additionally, many authors have reported multisystemic manifestations, such as in the central nervous system, retina, adrenal glands, hearing, and bones, similar to Zellweger syndrome [3,7,12,18]. However, in our case, the patient only presented with neurological symptoms, which made it difficult to diagnose. Hypoglycemia was reported as associated with the clinical onset in one D-BPD case by Werner et al. [20]. Their patient also presented desaturations, hypotonia, diminished reflexes, seizures, large fontanelle, and callosal hypoplasia. Our patient developed bilateral hydronephrosis grade II by 8 months, and callosal dysgenesis and acute obstructive hydrocephalus were noted at 11 months.

We could rule out Pompe disease and SMA by accessing free tests. However, not even routine genetic testing is not covered by the state Health Insurance House in Romania. As the infant’s seizures worsened and became more frequent and difficult to control, a non-governmental charity organization helped to conduct a genetic test for epilepsies, which also came back negative. Repeated cranial ultrasounds during the neonatal period showed non-specific aspects, prompting an MRI at two months. The MRI results suggested rare neurodegenerative disorders. The MRI scan was extremely helpful, providing crucial information, as there are reports of D-BPD cases with normal imaging aspects [3,20,35]. Germinolytic cysts are relatively common neuroimaging findings in D-BPD [4,18,35].

### 3.2. Genetic Diagnosis and D-BP Deficiency Classification

**Figure 3:**
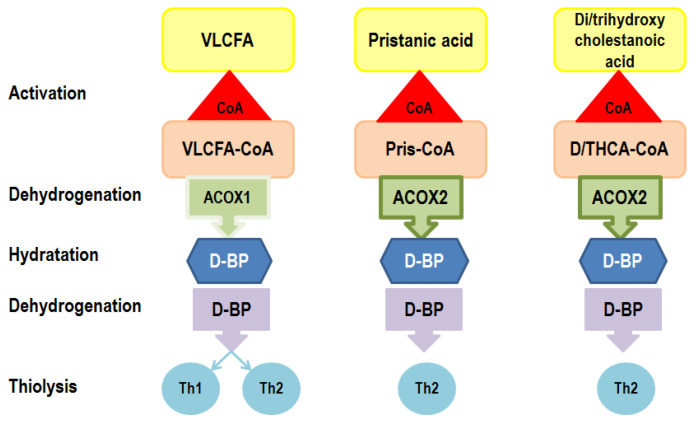
D-bifunctional protein peroxisomal enzymatic activity. Very-long-chain fatty acids (VLCFAs), pristanic acid (PRIS), dihydroxycholestanoic acid (DHCA), and trihydroxycholestanoic (THCA) are activated by free unesterified coenzyme A (CoA); acyl-CoA oxidase 1 (palmitoyl-CoA-oxidase) (ACOX1), and acyl-CoA oxidase 2 (branched-chain acyl-CoA oxidase) (ACOX2) are involved in the first step of peroxisomal β-oxidation, while D-bifunctional protein (D-BP) is involved in the next two steps—hydratation and dehydrogenation; straight-chain 3 oxoacyl-CoA thiolase (Th1) and sterol carrier protein-2/3-oxoacylCoA thiolase (sterol carrier protein X) (Th2) are involved in thiolysis [36,37].

D-bifunctional protein plays a crucial role in the peroxisomal fatty acids’ β-oxidation. Fatty acids β-oxidation consists of four reactions, two catalyzed by D-BP [2]. Two separate D-BP domains—3-hydroxyacyl-CoA dehydrogenase and 2-enoyl-CoA hydratase—are acting during the second and third step of peroxisomal fatty acids’ β-oxidation, leading to a shortage of fatty acid molecules (Figure 3). Conversion of the modified fatty acids in acetyl CoA follows, and acetyl CoA is transported outside the peroxisomes for reuse [3,4,9,16]. Variants of the *HSD17B4* gene, which encodes D-BP, may affect one or both D-BP functions and activity. The biochemical markers of D-BPD include the accumulation of very-long-chain fatty acids (C26:0), dihydroxycholestanoic acid, trihydroxycholestanoic acid, pristanic acid, and phytanic acid [3,9,18,34]. Fatty acid accumulation is detrimental to the developing central nervous system, leading to abnormal brain development and myelin decomposition, resulting in the loss of cerebral and medullar white matter (leucodystrophy) [10].

**Figure 4:**
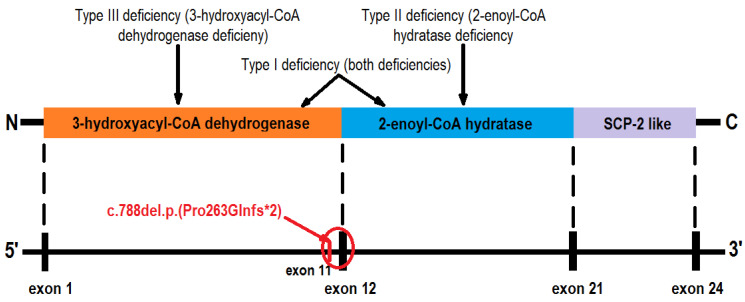
D-bifunctional protein domains, HSD17B4 gene schematic structure, and classification of D-BP deficiency; red—location of the described patient defect. Legend: SCP-2 like—steroid carrier protein-2 like.

The *HSD17B4* gene (601860) is responsible for oxidizing numerous peroxisomal substrates [19] and is located on chromosome 5 (5q.23.1). It is composed of 24 exons and 23 introns [3,16], with exons 1 to 12 coding for the N-terminal domain of 3-hydroxyacyl-CoA dehydrogenase, exons 12 to 21 coding for the 2-enoyl-CoA hydratase domain, and exons 21–24 being responsible for steroid-carrier protein 2-like (SCP-2 like) [3,16] (Figure 4). Homozygous and compound heterozygous mutations of the *HSD17B4* gene produce autosomal recessive D-BPD or Perrault syndrome [3,19]. Based on the enzymatic deficiency, D-BPD was classified into type I, where both 3-hydroxyacyl-CoA dehydrogenase and 2-enoyl-CoA hydratase are deficient, type II, where only hydratase is deficient, and type III, where only dehydrogenase is deficient [3,5,7,12,16]. In 2012, McMillan et al. [5] described D-BPD type IV as a less severe phenotype, like Perrault syndrome, due to missense mutations in both enzymatic domains leading to diminished but detectable protein activity. Recently, a type V of D-BPD was proposed, characterized by a less severe phenotype: neurosensorial hearing loss, mild intellectual disability, sensorimotor polyneuropathy, short stature, and ovarian dysgenesis (resembling Perrault syndrome) [17]. Some authors suggested that due to the rarity of D-BPD, the clinical and biochemical abnormalities that are encountered are not entirely understood [9,19].

Whole-exome sequencing revealed that our patient was homozygous for the variant *HSD17B4 c.788del*, p.(Pro263GInfs*2). The variant is absent in gnomAD but is reported in ClinVar. The defect is located on exon 12 and consists of the deletion of a base pair, generating a frameshift that prematurely stops codon2 amino acids downstream. The variant predicts either a truncation of the encoded protein or the absence of the protein, a common mechanism for disease [38]. Consequently, the patient was classified as having type I D-BPD, which matches its severe phenotype. Ferdinandusse et al. [9,11] proposed a genotype–phenotype correlation, as the depicted residual activity of D-BP was associated with less severe phenotypes in their cohort, and the mutation effect on the protein structure may predict the phenotype. This homozygous variant was previously described in 2006 in the cohort of Ferdinandusse et al. [9] and classified as pathogenic and associated with D-BPD (PMID 16385454). An unpublished observation in ClinVar reported an association of the same homozygous mutation in a patient with polymicrogyria, partial callosal agenesis, congenital hypotonia, epilepsy, developmental delay, dysmorphic features, anemia, and abnormal eye movement [38]. Other laboratories also detected the variant in the context of clinical testing (variation ID 632859 in ClinVar) [38]. Similarly, congenital hypotonia, epileptic seizures, polymicrogyria, callosal dysgenesis, and severe psychomotor delays were found in our patient.

The WES result showed that our patient was also homozygous for another variant, *SUOX c.913G>A, p.(Ala305Thr)*(NM_001032386), a variant with uncertain clinical significance in the ClinVar database. This missense mutation, localized on chromosome 12q13.2, is not expected to disrupt *SUOX* protein function. The variant is not present in gnomAD and has not been reported in the literature as being associated with conditions related to the *SUOX* gene. However, an entry was found in ClinVar (ID: 1427282) [38]. Polyphen cites the variant as possibly damaging and SIFT as tolerated, while MUTTASTER classifies the variant as disease-causing. Isolated deficiency of sulfite oxidase (ISOD), another rare, autosomal recessive neurometabolic disorder, has been classified into two types: a severe, classical form with neonatal onset and a milder form, with onset in infants and toddlers [39,40]. Unlike D-BPD, neonatal ISOD involves hypertonia, progressive spastic quadriplegia, muscular spasms leading to opisthotonus, and progressive microcephaly [39,40]. However, none of these symptoms were observed in our patient; therefore, the pathology associated with this variant in our patient, if any, was not found.

### 3.3. Considerations Facing an Atypical Presentation and in Low Resource Settings

**Figure 5:**
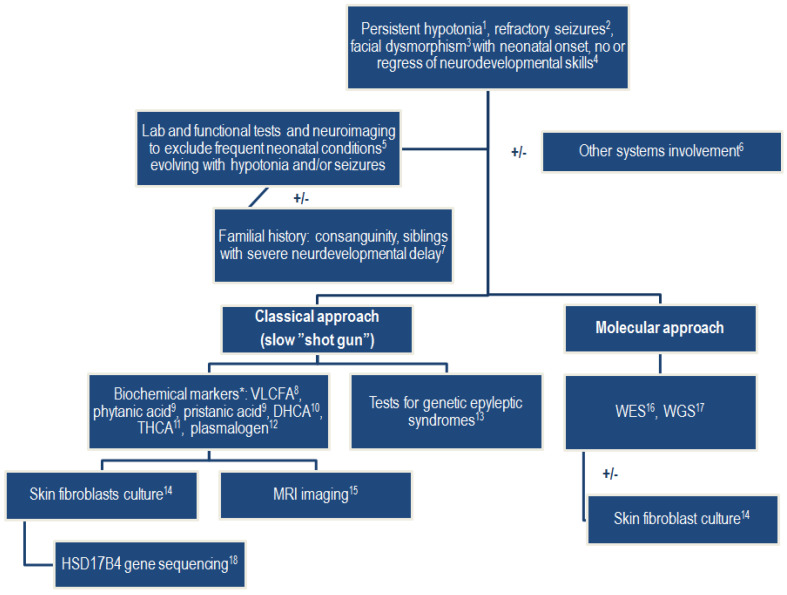
Diagnostic approach pathways in D-bifunctional protein deficit (D-BPD) during neonatal period. Legend: 1—hypotonia is present at birth in 98% of cases; 2—seizures occur at birth in 93% of cases and are refractory to anticonvulsants; 3—suggestive facial dysmorphism includes high forehead, hypertelorism, epicanthus, upslanted palpebral fissure, long philtrum, depressed nasal bridge, high arched palate, large fontanelle, retrognatism, macrocephaly, and low-set ears (most aspects are similar to Zellweger syndrome); 4—in severe forms of D-BPD, infants do not acquire any developmental skills; in mild forms, with some residual protein activity, infants may reach very early developmental milestones but gradual developmental regression is noted within a few months, and hyperreflexia and hypertonia develop with D-BPD progression; 5—routine biochemical tests, blood gases analysis, neonatal imaging (X-rays, ultrasound brain, heart, and abdomen), amplitude and conventional integrate electroencephalography, auditory and visual tests, DNA analysis that may exclude perinatal hypoxic–ischemic encephalopathy, neonatal sepsis and meningitis, chronic infections (TORCH syndrome), congenital brain abnormalities, and congenital neurological or muscular diseases; 6—D-BPD cases were reported in association with abnormal prenatal development (fetal ascites, polyhydramnios), other metabolic or homeostasis abnormalities (such as increased transaminases), digestive tract abnormalities (bile duct proliferation, cholestasis, hepatomegaly, hepatic steatosis, feeding difficulties), endocrine problems (primary adrenal insufficiency), renal cysts, splenomegaly, ocular abnormalities (nistagmus, strabismus, visual loss), osseous and muscular abnormalities (frontal bossing, dolichocephaly, clubfoot, hammertoe, split hand, clacific stippling, delayed skeletal maturation, osteopenia, decrease muscle mass), hearing impairments, central nervous system abnormalities (cerebral dysmyelination or hypoplasia, gliosis, cortical dysplasia, cerebellar atrophy, callosal hypoplasia or atrophy, ventriculomegaly), and failure to thrive; 7—consanguineous parents or siblings with neurodevelopmental delay (alive or dead) are suggestive of rare hereditary conditions; *—biochemical markers may be normal during neonatal period; 8—VLCFA—very-long-chain fatty acid levels are increased; 9—phytanic acid and pristanic acid (the final product of phytanic acid α-oxidation) are increased secondary to impaired peroxisomal β-oxidation due to D-BPD; 10—dihydroxycholestanoic acid (DHCA); 11—trihydroxycholestanoic acid (THCA); DHCA and THCA plasmatic levels are increased due to D-BPD; 12—normal plasmalogen levels in red blood cells excludes generalized peroxisomal disorders; 13—genetic testing may help exclude epileptic syndromes with neonatal/infantile onset; 14—cultures’ skin fibroblasts help in diagnosis in infants with normal biochemical markers during neonatal period and differential diagnosis from Zellweger syndrome, and allow for quantification of the enzyme activity, which is of further help in predicting D-BPD course; 15—magnetic resonance imaging may show congenital brain defects (see above; Legend 6), abnormal/delayed myelination, white substance demyelination, neuronal migration disorders, focal heterotopia, and germinolytic cysts; MRI may show normal aspects during the neonatal period, followed, in time, by a suggestive model of cerebral and cerebellar leukoencephalopathy; 16—WES—whole-exome sequencing; 17—WGS—whole-genome sequencing; 18—*HSD17B4 gene—17β-hydroxystroid dehydrogenase type 4* sequencing identifies the defect and its location and helps in predicting the patient’s outcome [2,3,4,5,6,7,8,9,10,12,15,16,18,19,20,28,34].

A typical and classic approach to D-BPD diagnosis involves several laborious steps [14,20] (Figure 5). The clinical suspicion is usually based on neonatal (even congenital) hypotonia, neonatal seizures, and facial dysmorphism [2,7,9,11,18]. Neuroimaging provides valuable information for the diagnosis, generally directed toward a neurodegenerative hereditary metabolic condition [4,18]. The next step involves measuring biochemical markers, followed by genetic testing, and finally, using skin fibroblast culture, direct measurement of the deficient enzyme and evaluation of C26:0 β-oxidation [2,5,9]. However, clinical aspects may not be suggestive; symptoms of the perinatal condition may overlap or may be similar to those of a rare disease, as early onset neonatal sepsis and perinatal hypoxia did in our patient. It is even more challenging to consider a genetic condition in the absence of physical markers that suggest a chromosomal abnormality.

Our patient did not show any obvious facial features suggesting a particular condition. The search for abnormal metabolic markers is difficult and expensive if one is not looking for a specific defect. The ability to search for metabolic markers may even be unavailable in low-resource settings. In our case, routine metabolic investigations consistently showed normal results. In Romania, free neonatal screening is only offered for congenital hypothyroidism, phenylketonuria, and, recently, cystic fibrosis. Stem cell banks offer extended metabolic screening for around 50 metabolic and endocrine hereditary conditions, and this is usually performed abroad, with parents having to pay for the tests. Unfortunately, not all families can afford the cost of such screenings or specific metabolic tests.

Our patient’s recurrent and refractory seizures prompted an evaluation for genetic epileptic syndromes, another time-consuming and expensive test. Neuroimaging in D-BPD may be normal during the neonatal period and during the follow-up [3,35,41]. In our case, neuroimaging was the first clue of a neurodegenerative disease. Fortunately, NGOs assist patients with limited financial resources, and in our patient’s case, they provided financial assistance for all expensive tests, including WES. Ultimately, WES clarified the diagnosis, offering valuable information on the disease and its outcome, and family counseling. The quantification of the enzymatic deficit using skin fibroblast cultures [2,3,4,9] was refused by the parents. However, there are also reports of normal VLCFA and phytanic acid in patients with D-BPD, a situation that may complicate diagnosis [19]. In D-BPD, WES offers a rapid diagnosis, and the genotype and defect’s location on the exon can predict the patient’s outcome [5,12,16].

On the other hand, with more accurate, rapid, and cheaper of WGS and WES, experts are calling for the use of these genetic tests at a larger scale, at least in critically ill children. Also, WES is considered essential in detecting conditions associated with heterogeneous phenotypes and genotypes and in cases with atypical clinical features [5]. A more rapid and precise diagnosis would, in this case, have broad implications for patient and resource management, helping both the patients and their families as an early diagnosis saves time, allows for the therapeutic strategies to be changed (if therapy is available) or for families to opt for palliation (if no treatment exists), and allows for family counseling for further reproduction and even the management of potentially affected siblings. Experts, on the other hand, emphasize that molecular diagnosis efficiently detects disease-causing mutations and structural variants in many disorders [14,22,23]. The costs of a molecular diagnosis must be balanced against the costs of a delayed correct diagnosis, prolonged hospitalization, re-admissions, evaluation in the outpatient clinic, multiple investigations, medications, and complications, and even the iatrogenic costs associated with multiple investigations [27,28,29]. Experts have recently recommended integrating genomic sequencing into the clinical activity of neonatal and pediatric intensive care units [30,42]. A national cohort study on newborns in Israel by Marom et al. [43] demonstrated that rapid trio-genome sequencing was feasible and diagnostically beneficial in public healthcare settings. Genomic sequencing provides an early and precise diagnosis, particularly useful in newborns with hereditary conditions and clinical situations where traditional testing approaches may fail due to overlapping symptoms during the neonatal period. Early genomic sequencing offers several advantages, including immediate precision medicine for curable conditions through timely and targeted interventions, significant improvements in neonatal intensive care, and familial counseling [42]. Even in low-resource settings and settings without specialists in rare hereditary diseases, genetic sequencing should be considered for essential investigations in the face of atypical and critical cases. Our experience with the presented case and many other cases supports this recommendation.

## 4. Conclusions

This paper provides an overview of the clinical, imaging, biochemical, and molecular data and describes the clinical course of the first Romanian patient diagnosed with D-BPD. We highlight the challenges faced in using the traditional diagnosis approach for a patient with an incomplete clinical picture of D-BPD and common neonatal symptoms in a low-resource setting. Our conclusions concur with the recent expert recommendations that genome sequencing integration is a prompt and efficient diagnostic tool for critically ill infants in neonatal and pediatric intensive care units.

## Data Availability

Data are contained within the article and Supplementary Materials.

## Supplementary Materials

The following supporting information can be downloaded at: https://www.mdpi.com/article/10.3390/ijms25094924/s1.
