# Supplementary material for: D-Bifunctional Protein Deficiency Diagnosis—A Challenge in Low Resource Settings: Case Report and Review of the Literature
Source: Int J Mol Sci. 2024 Apr 30;25(9):4924. doi: 10.3390/ijms25094924 (PMC11084724; doi:10.3390/ijms25094924)
Supplement: Supplementary file 1 [file ijms-25-04924-s001.zip › ijms-2937459-supplementary.pdf]

Supplementary material. Technical notes of the Whole Exome Sequencing

Whole exome sequencing was carried out by a commercial laboratory (Blueprint Genetics, Finland), using their Blueprint Genetics Whole Exome Plus Test (version 2, Feb 9, 2018). This assay consists of sequence analysis of all protein-coding genes in the genome for the proband, coupled with Whole Exome Deletion/Duplication (CNV) Analysis. The test targets all protein-coding exons, exon-intron boundaries (± 20 bps), and selected noncoding, deep intronic variants (listed in the Summary of the test). This test is used to detect single nucleotide variants and small insertions and deletions (INDELs) up to 220 bps and copy number variations defined as single exon or larger deletions and duplications. This test is not used to detect repeat expansion disorders or diseases caused by mitochondrial DNA (mtDNA) mutations. The test does not recognize balanced translocations or complex inversions and may not detect low-level mosaicism.

The Whole Exome Plus Test analysis is primarily focused on established disease genes previously associated with genetic disorders. The genes with known clinical association include those curated by Blueprint Genetics (BpG) and included in BpG diagnostic panels (>4140 genes). These genes are supplemented with genes included in The Clinical Genomics Database (>4320 genes) and the Developmental Disorders Genotype-Phenotype Database (DD2GP) (>2190 genes). The total number of genes considered clinically associated in the Whole Exome Plus analysis is >4780 (and the number is constantly updated). If analysis of exome variants in previously established disease genes is inconclusive, exome variant data are also analyzed for variants not located within known clinically associated genes but have properties that make them candidates for potentially disease-causing variants (please see also Summary of the Test). If, over time, other patients with a similar phenotype and variants in the same gene are identified, the variant may be reclassified as a likely cause of the disorder.

Whole-exome sequence analysis of variants in previously established disease genes

Given that the patient has no reported family history of the disease, the exome data of the patient was analyzed for rare heterozygous variants (potential de novo variants) and variants following recessive inheritance patterns. Sequence analysis using the Blueprint Genetics (BpG) Whole Exome Plus identified a homozygous frameshift variant *HSD17B4* c.788del, p.(Pro263Glnfs*2) and a homozygous missense variant *SUOX* c.913G>A, p.(Ala305Thr).

Secondary findings. The patient opted in for an analysis of secondary findings, which are sequence variants unrelated to the indication for ordering the sequencing but of medical value for patient care. The patient's Whole Exome data was analyzed for secondary findings according to the recommendations of the American College of Medical Genetics and Genomics (ACMG; PMID: 27854360).

The analysis was negative for secondary findings.

Coverage Plot – nuclear genes.

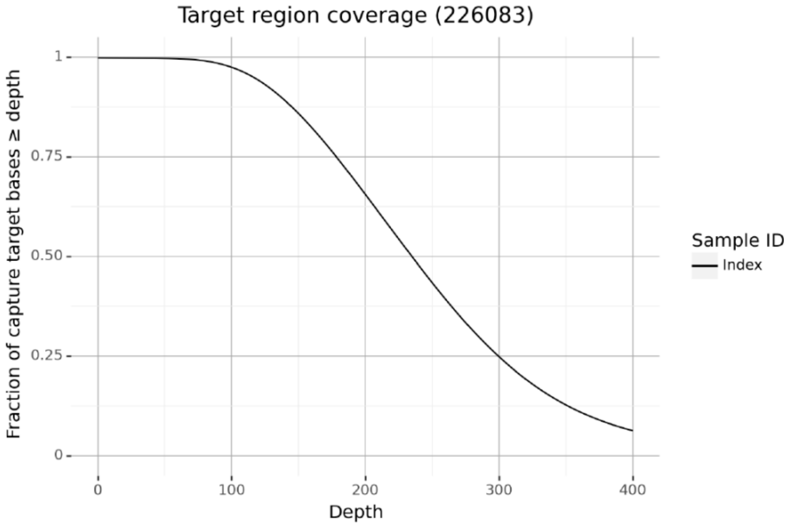

Summary of the Test- Whole exome

1.1. Laboratory process: The total genomic DNA was extracted from the biological sample using a bead-based method when required. The quantity of DNA was assessed using the fluorometric method. After assessing DNA quantity, a qualified genomic DNA sample was randomly fragmented using non-contact, isothermal sonochemistry processing. The sequencing library was prepared by ligating sequencing adapters to both ends of DNA fragments. Sequencing libraries were size-selected with a bead-based method to ensure optimal template size and amplified by polymerase chain reaction (PCR). Regions of interest (exons and intronic targets) were targeted using a hybridization-based target capture method. The quality of the completed sequencing library was controlled by ensuring the correct template size and quantity and eliminating the presence of leftover primers and adapter-adapter dimers. Ready sequencing libraries that passed the quality control were sequenced using Illumina's sequencing-by-synthesis method using paired-end sequencing (150 by 150 bases). Primary data analysis, which converts images into base calls and associated quality scores, was carried out by the sequencing instrument using Illumina's proprietary software, generating CBCL files as the final output.

1.2. Bioinformatics and quality control: Base-called raw sequencing data has been transformed into FASTQ format using Illumina's software (bcl2fastq). Sequence reads of each sample were mapped to the human reference genome (GRCh37/hg19). Burrows-Wheeler Aligner (BWA-MEM) software was used for read alignment. Duplicate read marking, local realignment around indels, base quality score recalibration, and variant calling were performed using GATK algorithms (Sentieon) for nDNA. Variant data was annotated using a collection of tools (VcfAnno and VEP) with a variety of public variant databases, including but not limited to gnomAD, ClinVar, and HGMD. The median sequencing depth and coverage across the target regions for the tested sample were calculated based on MQ0-aligned reads. The sequencing run included in-process reference sample(s) for quality control, which passed our thresholds for sensitivity and specificity. The patient's sample was subjected to thorough quality control measures, including contamination and sample mix-up assessments. Copy number variations (CNVs), defined as single exon or larger deletions or duplications (Del/Dups), were detected using a proprietary bioinformatics pipeline from the sequence analysis data. The difference between observed and expected sequencing depth at the targeted genomic regions was calculated, and regions were divided into segments with

variable DNA copy number. The expected sequencing depth was obtained using other samples processed in the same sequence analysis as a guiding reference. The sequence data was adjusted to account for the effects of varying guanine and cytosine content.

1.3. Interpretation: The pathogenicity potential of the identified variants was assessed by considering the predicted consequence, the biochemical properties of the codon change, the degree of evolutionary conservation, as well as several reference population databases and mutation databases such as, but not limited to the 1000 Genomes Project, gnomAD, ClinVar, and HGMD Professional. For missense variants, *in silico* variant prediction tools such as SIFT, PolyPhen, and MutationTaster are used to assist with variant classification. In addition, the clinical relevance of any identified CNVs was evaluated by reviewing the relevant literature and databases such as 1000 Genomes Project, Database of Genomic Variants, ExAC, gnomAD, and DECIPHER. The clinical evaluation team assessed the pathogenicity of the identified variants by evaluating the information in the patient referral, reviewing the relevant literature, and manually inspecting the sequencing data if needed. Reporting was performed using HGNC-approved gene and mutation nomenclature following the HGVS guidelines. Likely benign and benign variants are not reported. In addition to the analysis of variants in previously established disease genes, variants in genes where disease association has not yet been established were considered as potentially disease-causing

mainly using the following scheme: a) for probands who were whole-exome sequenced with parents, all coding region de novo variants were evaluated; b) novel (absent in gnomAD) heterozygous, truncating variants (nonsense, frameshift, canonical splice site variants) in genes predicted to be intolerant for loss-of-function variation based on gnomAD variant data. Genes are determined as intolerant if the probability of loss-of-function intolerance score (pLI) is ≥0.9. The closer the pLI is to one, the more LoF intolerant the gene appears. Genes with pLI≥0.9 are defined as an extremely LoF intolerant set of genes; c) rare (<1% MAF in gnomAD), truncating homozygous or (predicted) compound heterozygous variants, or a combination of rare truncating and rare missense variant that is predicted deleterious by multiple *in silico* tools; d) in addition, only variants in genes whose known expression pattern and function are considered relevant for the phenotype are included. For proband and family members who opted to analyze secondary findings from the WES data, clinically actionable genes are analyzed and reported for secondary findings according to recommendations by ACMG (PMID 27854360). Variants within ACMG genes associated with autosomal dominant phenotypes classified as pathogenic or likely pathogenic are reported. Genes associated with phenotypes inherited in an autosomal recessive manner need two variants classified as likely pathogenic/pathogenic (or a homozygous variant) to meet the threshold for reporting. Pathogenic and likely pathogenic variants within genes associated with X-linked phenotypes that are apparently hemizygous, heterozygous, compound heterozygous, or homozygous are reported. The analysis of secondary findings does not include an analysis of CNV data. Secondary findings are not analyzed or reported for deceased individuals or fetal samples.

1.4. Variant classification: Our variant classification follows the Blueprint Genetics Variant Classification Schemes modified from the ACMG guideline 2015. Minor modifications were made to increase the reproducibility of the variant classification and improve the clinical validity of the report. The classification and interpretation of the variant(s) identified reflect the current state of Blueprint Genetics' understanding at the time of this report. Variant classification and interpretation are subject to professional judgment and may change for various reasons, including but not limited to updates in classification guidelines and the

availability of additional scientific and clinical information. This test result should be used with the health care provider's clinical evaluation. Inquiries regarding potential changes to the variant's classification are strongly recommended before making future clinical decisions.

1.5. Confirmation of sequence alterations: Sequence variants classified as pathogenic, likely pathogenic, and variants of uncertain significance (VUS) were confirmed using bi-directional Sanger sequencing when they did not meet our stringent NGS quality metrics for a true positive call.

1.6. Confirmation of copy number variants: CNVs (Deletions/Duplications) are confirmed using a digital PCR assay if they covered less than 10 exons (heterozygous), less than 3 exons (homo/hemizygous) or were not confirmed at least three times previously at our laboratory. Furthermore, CNVs of any size are not confirmed when the breakpoints of the call could be determined.

1.7. Analytic validation: Blueprint Genetics has independently validated this laboratory-developed test. The sensitivity of this panel is expected to be in the same range as the validated whole exome sequencing laboratory assay used to generate the panel data (sensitivity for SNVs 99.65%, and indels 1-50 bps 99.07%, one-exon deletion 92.3% and two exons CNV 100%, and specificity >99.9% for most variant types). It does not detect very low-level mosaicism, as a variant with a minor allele fraction of 14.6% can be detected in 90% of the cases.

1.8. Test restrictions: A normal result does not rule out the diagnosis of a genetic disorder since some DNA abnormalities may be undetectable by the applied technology. Test results should always be interpreted in the context of clinical findings, family history, and other relevant data. Inaccurate or incomplete information may lead to the misinterpretation of the results.

1.9. Technical limitations: This test does not detect the following: complex inversions, gene conversions, balanced translocations, repeat expansion disorders unless specifically mentioned, and non-coding variants deeper than ±20 base pairs from exon-intron boundary unless otherwise indicated. Additionally, this test may not reliably detect the following: low-level mosaicism, stretches of mononucleotide repeats, indels larger than 50bp, single exon deletions or duplications, and variants within pseudogene regions/duplicated segments. The sensitivity of this test may be reduced if DNA is extracted by a laboratory other than Blueprint Genetics. Laboratory error is also possible.

1.10. Regulation and accreditations: Blueprint Genetics developed This test and its performance characteristics. The US Food and Drug Administration has not cleared or approved it. This analysis has been performed in a CLIA-certified laboratory (#99D2092375), accredited by the College of American Pathologists (CAP #9257331) and by FINAS Finnish Accreditation Service (laboratory no. T292), accreditation requirement SFS-EN ISO 15189:2013. All the tests are under the scope of the ISO 15189 accreditation. The sample was analyzed using CE marked Blueprint Genetics CES Platform and/or Blueprint Genetics WES Platform in vitro diagnostic medical device manufactured by Blueprint Genetics Oy.

Legend: gnomAD = genome Aggregation Database (reference population database; >138,600 individuals); MutationTaster = *in silico* prediction tools used to evaluate the significance of identified amino acid changes. PolyPhen = *in silico* prediction tool used to evaluate the significance of amino acid changes; SIFT = *in silico* prediction tool used to evaluate the significance of amino acid changes.
